# Investigating the Signature of Aquatic Resource Use within Pleistocene Hominin Dietary Adaptations

**DOI:** 10.1371/journal.pone.0069899

**Published:** 2013-08-21

**Authors:** Will Archer, David R. Braun

**Affiliations:** 1 Human Evolution Department, Max Planck Institute of Evolutionary Anthropology, Leipzig, Germany; 2 Archaeology Department, University of Cape Town, Cape Town, South Africa; 3 George Washington University, Center for the Advanced Study of Hominin Paleobiology, Washington DC, United States of America; University of Florence, Italy

## Abstract

There is general agreement that the diet of early hominins underwent dramatic changes shortly after the appearance of stone tools in the archaeological record. It is often assumed that this change is associated with dietary expansion to incorporate large mammal resources. Although other aspects of the hominin diet, such as aquatic or vegetal resources, are assumed to be a part of hominin subsistence, identifying evidence of these adaptations has proved difficult. Here we present a series of analyses that provide methodological support for the inclusion of aquatic resources in hominin dietary reconstructions. We suggest that bone surface modifications in aquatic species are morphologically distinguishable from bone surface modifications on terrestrial taxa. We relate these findings to differences that we document in the surface mechanical properties of the two types of bone, as reflected by significant differences in bone surface microhardness values between aquatic and terrestrial species. We hypothesize that the characteristics of bone surface modifications on aquatic taxa inhibit the ability of zooarchaeologists to consistently diagnose them correctly. Contingently, this difficulty influences correspondence levels between zooarchaeologists, and may therefore result in misinterpretation of the taphonomic history of early Pleistocene aquatic faunal assemblages. A blind test using aquatic specimens and a select group of 9 experienced zooarchaeologists as participants was designed to test this hypothesis. Investigation of 4 different possible explanations for blind test results suggest the dominant factors explaining patterning relate to (1) the specific methodologies employed to diagnose modifications on aquatic specimens and (2) the relative experience of participants with modifications on aquatic bone surfaces. Consequently we argue that an important component of early hominin diets may have hitherto been overlooked as a result of (a) the paucity of referential frameworks within which to identify such a component and (b) the inability of applied identification methodologies to consistently do so.

## Introduction

### Research impetus

The reconstruction of Pleistocene hominin diet is a field that has major implications for the mechanisms that shaped the evolutionary history of our lineage. However, evidence for variability in hominin diets is difficult to ascertain. Currently evidence of hominin diet can be extrapolated from only a few sources. Isotopic composition of dental enamel, dental microwear and trace fossils recovered from dental calculus provide some of the main indicators e.g.[1], [2], [3]. Other than these measures, inferences about hominin diet can be derived from butchery marks on the surfaces of bones of potential prey of hominins [4]. However the vast majority of bone surface modification studies are conducted on the bones of large terrestrial mammals. Often there has been an assumption that these animals were the major prey items of hominins [5]. However, recently the role of small animals and aquatic resources in hominin diet has been investigated [6], [7], [8]. In a previous study of aquatic remains from the site of FwJj20 in northern Kenya, modifications on aquatic animals were recognized to be different in size and shape from modifications on terrestrial fauna. This finding has considerable implications for other early Pleistocene localities containing unstudied aquatic fauna.

Indeed, many early Pleistocene faunal assemblages contain a substantial component of aquatic animals. However, compared with well-studied terrestrial components, relatively little is known about the role of aquatic resource exploitation within hominin subsistence at this time. This stands in stark contrast to abundant literature discussing the role of large terrestrial mammalian carcasses in Early Pleistocene hominin adaptation e.g. [9]–[13, and many others]. Stewart [14] first emphasized the potential importance of aquatic resources to early hominin diets, and this has been echoed by a number of recent studies [15]–[18], [8], [19].

Aquatic resource access represents a key adaptive shift within the expansion of hominin subsistence bases and increases in hominin dietary quality [20]. This shift likely equated to increasing the overall proportion of fats and proteins hominins consumed [21], [14]. Recent reviews of hominin dietary requirements have emphasized the inability of hominin biology to withstand dramatic variations in dietary resources [22]. These reviews question the importance of large terrestrial animals as a key resource in hominin diet because of the dramatic seasonal fluctuations in dietary quality of these resources [22], [23], [24].

A substantial challenge to investigating (1) the timing of the above mentioned dietary shift and (2) the dietary importance of aquatic resources within human evolutionary history is the diagnosis of material traces of aquatic resource exploitation, such as unequivocal surface modifications on the bones of aquatic animals. Surface modifications provide a behavioral link between the numerous fossils of aquatic animals and other archaeological materials e.g. [7], [25]. Establishment of this inferential link would indicate that reconstructions of early Pleistocene hominin diet that focus *only* on terrestrial components could be missing a crucial element of hominin subsistence.

Evidence for the butchery and consumption of aquatic resources based on surface modification data is limited even in younger contexts. Holocene and late Pleistocene assemblages document the difficulty with identifying diagnostic features of hominin modification [26]. However, the abundance of fish remains as well as associated material culture (e.g. hooks, harpoons and net weights) are usually sufficient in late Pleistocene contexts to establish a behavioral association between hominins and aquatic resources [27]. Ethnographic studies have documented that when definitive aquatic resource processing is observed, modifications on fish bones are rare [28]. Further, experimental studies have investigated the likelihood that the processing of aquatic remains resulted in diagnostic traces on bones. These experiments documented the location and frequency of marks that were butchered with stone artefacts [29]. Yet despite these experimental and ethnographic studies, the identification of associated cut-marks on fish or other aquatic resources is rare in the archaeological record [28], [29], [14].

Given these difficulties with identifying modifications on all aquatic bone it is unclear whether the few reports of aquatic resource consumption in early Pleistocene contexts represent early isolated events, or whether a shift in applied methodology might reveal these results to reflect widespread subsistence behaviors. Indeed the explanation for the relative rarity of aquatic bone surface modifications in early Pleistocene contexts where associated material evidence of aquatic resource use is lacking, remains unknown. Given the scale and rigor with which the Early Pleistocene zooarchaeological record has been approached over the last two decades [30], [13], [31]–[35], [10], [36]–[43], if these marks were present and reliably identifiable, it seems likely that they would be routinely reported if aquatic resources were a substantial part of early Pleistocene hominin diets. Yet surface modifications on aquatic resources are *rarely* reported [14], [44], [45], even when aquatic animals appear to be abundant in archaeofaunas.

### Research framework

The interpretive capacity of surface modification data generally is determined by two prerequisites [4]. First, marks must be identifiable to both actor (e.g., carnivore or hominin) and effector (e.g., tooth, flake etc.). In general, there needs to be relatively high inter-analyst agreement on mark identification for frequency estimations based on these identifications to be meaningful [4]. Secondly, interpretations of mark frequencies should be based on highly resolved experimental models where actor and effector are both known [4]. Blumenschine and colleagues [4] argue further that even inconspicuous marks on terrestrial bones can be identified to actor and effector if specific conditions are met. These conditions include the necessity to have analysts with extensive experience and *suitable* control collections where actors and effectors are known.

Blumenschine et al.'s [4] data indicates that despite the substantial body of research on cut-mark mimicry and overlap in different mark type morphologies [46]–[51], surface modifications on terrestrial mammalian remains can be distinguished reliably by experienced analysts.

Here we investigate the applicability of previous bone surface modification identification protocols for identifying stone tool use in the processing of aquatic fauna. We describe an experimental dataset of aquatic fauna with known modifications and investigate whether correct diagnosis of actor and effector by nine experienced zooarchaeologists fulfills the requirements for reliable identification. We develop an experimental framework to (1) investigate why indicative traces of early Pleistocene aquatic resource exploitation may have been under-reported in the past and to (2) identify methodological parameters within which these traces can be accurately identified and diagnosed in the future.

We present results from a blind test structured broadly on the format described by Blumeschine et al. [4]. The blind test was designed to (a) evaluate the overall accuracy of identifying known marks to actor and effector; (b) determine the correspondence between analysts in accurate mark identification; (c) compare the ambiguity in aquatic identifications to results obtained in tests where only terrestrial specimens were used; and (d) evaluate the effects of various internal as well as external influences on the results of (a) and (b).

This study confirms the unique character of modifications on aquatic taxa and indicates that the current protocol for identifying these marks results in incorrect diagnoses and under-reporting. Our experiments suggest that the frequent use of a 10× hand lens to identify surface modifications is not adequate to accurately identify marks on aquatic bone surfaces at frequencies that are behaviorally meaningful.

We also investigate various hypotheses regarding *why* identification correspondence and success rates on actor diagnosis among surface modifications on aquatic taxa are relatively low in the current reported blind tests.

We recognize that there are numerous external factors that potentially affect blind-test results. We explore a number of these factors that could theoretically be driving correspondence scores and success rates including the effect of (1) pedagogical pedigree or teaching tradition, (2) participant experience depth and (3) the inclusion of certain types of surface modifications related to sedimentary abrasion which are particularly susceptible to misidentification [47], [49]. Finally we also interrogate variability in aquatic modification shape and size as an explanation for their misidentification. Further we investigate differences in the micro-hardness of bone surfaces as an explanation for the morphology of aquatic modifications.

We suggest that mechanical and morphological properties of aquatic bone results in surface modifications that are often qualitatively and quantitatively distinguishable from those made on mammalian bones. Our results suggest that participating analysts identified aquatic surface modifications using a referential framework that was based on terrestrial mammalian bones. In consequence, these analysts did not readily recognize and easily diagnose modifications produced on bone surfaces of aquatic animals. This may explain why identification accuracy and inter-analyst correspondence rates are generally low on aquatic bone specimens.

Our findings suggest that for surface modification frequencies on aquatic archaeological bone to reach their maximum potential for paleoanthropological interpretation (1) their initial identification needs to be approached differently to terrestrial modifications and (2) analysts making identifications need to refer to a directly applicable aquatic referential framework when doing so.

These methodological considerations may explain why a potentially important component of hominin diet remains relatively poorly understood.

## Methods

### Ethics statement

1. **No permits were required for the described study, which complied with all relevant regulations in the countries in which the described research was conducted.**
2. The terrestrial and aquatic vertebrate subjects (*Clarias* catfish as well as *Raphicerus Campestris* and *Ovis Aries*) of these experiments were already dead when they were purchased. Therefore the act of killing was not factored into the decision to use these individuals for research purposes. The aquatic specimens were purchased legally from Kenyan fisherman at Lake Turkana. The terrestrial specimens were purchased from a licensed butcher in the Western Cape Province, South Africa.
3. We state categorically that **the procurement and killing of these animals were not components of the controlled experiments reported in this manuscript**. **No animals used in this study were injured or killed specifically for research purposes.**

### Experimental datasets

Specimens used in the blind test were selected from 21 fish butchery experiments. These experiments included 17 large catfish (*Clarias gariepinus*), a Nile Perch (*Lates longispinis*) and a medium sized cichlid fish (*Oreochromis niloticus*). The series of experiments focused on catfish butchery as various species of the family *Claridae* are common in aquatic faunal assemblages in many ethnographic as well as archaeological assemblages in East Africa [14], [52]. Fish were procured from local fishermen on the eastern shores of Lake Turkana in the Marsabit District of northern Kenya. The butchery and trampling experiments were carried out in East Turkana, Northern Kenya over a six week period in June–July 2011. All specimens were butchered by a single adult male, from the Turkana tribe, who had extensive experience with the processing of aquatic resources.

Unretouched stone flakes made from basalt and ignimbrite (raw materials utilized by the hominins that produced the archaeological assemblages from the Koobi Fora Formation [53], [54]) were used for skinning and defleshing each carcass. These flakes were selected based on their similarity in size and shape to those recovered from the early Pleistocene archaeological assemblage of FwJj 20 [8]. For the processing of each carcass, a single flake was utilized in order to standardize the morphology of the marks produced in each experiment. A specific length of edge was designated for use on each flake (100 mm) and the mean edge angle of the designated edge was restricted within a 15 degree range.

The fisherman followed broadly the same disarticulation and flesh-removal sequence for each catfish butchered. This entailed: (1) Laying the fish on its side or ventral surface, with the caudal fin facing away from the butcher. (2) The pectoral fin was generally held and lifted to stabilize the subject. (3) A deep forceful incision was made roughly perpendicular or slightly diagonal to the antero-posterior axis of the fish. This incision sliced through the entire proximal diameter of the fillet. This incision was generally close to the posterior cranial bones and the flake often visibly made contact with the post-temporal or supracleithrum. (4) A series of shorter delicate slices are made perpendicular to, but in close proximity to the incision mentioned in “(3)”. These strokes serve to lift the proximal end of the fillet muscle mass off the neural spines of the cervical vertebrae. This ensured that the maximum width of fillet available can be removed with subsequent slices. (5) The butcher then removed his hand from the pectoral fin and held the proximal end of the fillet, pulling it gently away from the vertebral column to increase the tension on the fillet flesh attached to the vertebral column. (6) The butcher often put their foot on the fish's head or co-opted assistance from another fisherman do so. This freed the hand that had been holding the pectoral fin. (7) A sequence of short strokes towards the caudal region ensued, that severed the entire fillet from the vertebral column and ribs. This sequence of alternating slices was often associated with the fisherman's other hand tearing the fillet off the vertebral column. Here the flake occasionally made contact with, and sliced through both neural and haemal spines on the vertebra. The process of de-fleshing of the cranium was more variable, but resulted in multiple contacts between tool edges and various elements of the skull. Hammerstones were sometimes used for accessing fatty meat inside the neurocranium of the catfish specimens and were also occasionally used to kill catfish immediately after they were caught. Many catfish species have an external breathing accessory; as such they can survive outside of water for substantially longer than other fish. Consequently they are often killed by fishermen immediately after being caught, a behavior that results in percussion damage on cranial fragments.

All aquatic butchery experiments were conducted under the supervision of the senior author. Photos of the different butchery activities were taken at multiple intervals during the butchery process. Stroke count and time taken to complete specific tasks within each butchery episode were recorded when and where feasible (Table 1).

**Table 1:** Stroke count and time taken to complete phases of butchery for catfish and crocodiles.

Experiment number(Clarias)	Live weight (kg)	Strokes filleting	Strokes defleshing head	Time filleting (minutes)	Time defleshing head (minutes)
**6**	6	529	622	8	11
**7**	7.2	855	702	10	15
**9**	3.2	848	454	11	9
**11**	4.5	608	470	7.3	7
**12**	2	708	240	11	4.3
**13**	4	1170	430	14	6
**15**	2.1	350	180	10	10

Experiment number(Large reptiles)	Weight (kg)	Strokes skinning	Strokes defleshing	Time skinning	Time defleshing
**10**	5	674	1113	11	20
**14**	4	728	610	15	14

The experimental assemblage from which cross-sectional measurements discussed below were calculated (*2.4*), comprise a sample of sixty two cut-marks, including a terrestrial sample from a previous series of butchery experiments (Table 2). All mammalian surface modifications were collected from two adult butchered size 1 bovids. Tool dimensions and stroke count were controlled within the mammalian butchery experiments (Table 2). The mammalian cut-marks measured varied in terms of what surfaces (cancellous, thinning cortical and cortical) and portions (mid-shaft, near epiphyseal and epiphyseal) measured cut-marks appeared on (Table 2). The aquatic sample on which cross-sectional morphology was measured (*2.4*) derive from the same aquatic butchery experiments described above.

**Table 2:** Terminology under ‘Portion’ after Blumenschine (1995), MSH: Mid-shaft fragment, NEF: Near epiphyseal fragment and EPIPH: Epiphyseal.

Specimen	Species	Element	Portion	Surface	Tool edge type	Mean edge angle	Tool mass
**20a**	*Raphicerus Campestris*	Radius	NEF	thinning cortical	unmodified	61.7	29 g
**20b**	*Raphicerus Campestris*	Radius	NEF	thinning cortical	unmodified	61.7	29 g
**20c**	*Raphicerus Campestris*	Radius	EPIPH	cancellous	unmodified	61.7	29 g
**20d**	*Raphicerus Campestris*	Radius	EPIPH	cancellous	unmodified	61.7	29 g
**22a**	*Raphicerus Campestris*	Humerus	MSH	cortical	unmodified	61.7	29 g
**22b**	*Raphicerus Campestris*	Humerus	MSH	cortical	unmodified	61.7	29 g
**23a**	*Raphicerus Campestris*	Femur	NEF	thinning cortical	unmodified	57.5	50.2 g
**23b**	*Raphicerus Campestris*	Femur	NEF	thinning cortical	unmodified	57.5	50.2 g
**24a**	*Raphicerus Campestris*	Metacarpal	EPIPH	cancellous	unmodified	57.	50.2 g
**24b**	*Raphicerus Campestris*	Metacarpal	EPIPH	cancellous	unmodified	57.5	50.2 g
**24c**	*Raphicerus Campestris*	Metacarpal	EPIPH	cancellous	unmodified	57.5	50.2 g
**24e**	*Raphicerus Campestris*	Metacarpal	MSH	cortical	unmodified	57.5	50.2 g
**24f**	*Raphicerus Campestris*	Metacarpal	MSH	cortical	unmodified	57.5	50.2 g
**25f**	*Raphicerus Campestris*	Calcaneum	EPIPH	cancellous	unmodified	57.5	50.2 g
**2a**	*Raphicerus Campestris*	Metacarpal	NEF	thinning cortical	unmodified	57.5	50.2 g
**2d**	*Raphicerus Campestris*	Metacarpal	MSH	cortical	unmodified	57.5	50.2 g
**2e**	*Raphicerus Campestris*	Metacarpal	NEF	thinning cortical	unmodified	57.5	50.2 g
**3a**	*Raphicerus Campestris*	Tibia	NEF	thinning cortical	unmodified	63.3	33.2 g
**19a**	*Ovis Aries*	Femur			bifacial	69.9	160 g
**004a**	*Ovis Aries*	Radius	NEF	thinning cortical	bifacial	72.4	53.3 g
**004b**	*Ovis Aries*	Radius	NEF	thinning cortical	bifacial	72.4	53.3 g
**005a**	*Ovis Aries*	Humerus	EPIPH	cancellous	bifacial	72.4	53.3 g
**005b**	*Ovis Aries*	Humerus	EPIPH	cancellous	bifacial	72.4	53.3 g
**005c**	*Ovis Aries*	Humerus	EPIPH	cancellous	bifacial	72.4	53.3 g
**005d**	*Ovis Aries*	Humerus	MSH	cortical	bifacial	72.4	53.3 g
**005e**	*Ovis Aries*	Humerus	MSH	cortical	bifacial	72.4	53.3 g
**006a**	*Ovis Aries*	Rib			bifacial	61.2	383 g
**007a1**	*Ovis Aries*	Rib			bifacial	61.2	383 g
**007b1**	*Ovis Aries*	Rib			bifacial	61.2	383 g
**007c**	*Ovis Aries*	Rib			bifacial	61.2	383 g
**008a**	*Ovis Aries*	Metatarsal	NEF	thinning cortical	bifacial	69.9	160 g

Terminology under ‘Surface’ after Selvaggio and Wilder (2001).

### Blind-test assemblage

To develop a blind test that included a wide range of actors and effectors, the aquatic bones were subjected to a variety of processes that produce surface modifications. These include:

- Percussion damage: Modifications to the neurocranium of the catfish resulted from two processes. The first process was fracturing of the neurocranium which is integrated into the butchery activity as described above. The second was associated with the fracture of the neurocranium conducted by butchers to kill the catfish during or shortly after the fish are caught. Marks from these activities derive from the application of dynamic load with a rounded and occasionally angular hammerstone.
  Percussion damage associated with tissue acquisition occurs elsewhere on the carcass, predominantly at or in close proximity to sutures on the neurocranium. Percussive activities resulted in clusters of striations and occasionally micro-fractures that emanate from the point of contact. At suture lines these fractures usually run oblique to the surface of the bone in a similar fashion to those described for percussion fracture of tortoise carapaces [7].
- Cut and scrape marks: The butchery process involved the full removal of all tissues from the carcass. This included filleting of the large muscle masses on the body of the fish as well as the disarticulation of many of the bones from the head of the catfish. Observed contact between tool edges and bone surfaces were noted during the butchery process so it was occasionally possible to reconstruct activities associated with mark production. Cut and scrape marks are caused during observed episodes of filleting and flesh removal. Marks resulting from cutting and scraping were usually isolated in areas with large amounts of flesh adhering to bone surfaces. In particular, the removal of flesh from the inside of the catfish neurocranium and the other associated bony elements of the catfish head (e.g. cleithrum) resulted in frequent tool-bone contact. Macroscopically these appear to be groups of linear striations. On fresh bone surfaces these are often difficult to identify even when the locations of modifications are known.
- Tooth marks: None of the bones in this experiment were exposed to large mammal or reptilian carnivore ravaging. Many of the bones in the experiments were considered to be small enough that any type of carnivore ravaging would have deleted these elements entirely. Two specimens were eaten by humans without the assistance of cutlery which resulted in human tooth marks on a number of elements (which were correctly identified as such by several blind test participants). Human tooth marks have been documented in several actualistic and archaeological contexts [7], [55], [56]. Recently, the presence of human tooth marks has been noted on other aquatic assemblages in archaeological contexts [7]. These marks can be distinguished by their small size and their association with “peeling” damage and crushed edges of bones [57].
- Trampling damage: The linear striations caused by sedimentary abrasion can often produce marks that mimic hominin butchery activities [47], [49]. To simulate these effects two cleithrum bones, which did not have butchery marks on them (these specimens were collected from the numerous freshly killed catfish that are discarded near modern fishing encampments), were trampled. These specimens were placed on a coarse sand matrix, which contained a small number of unmodified sub-angular and rounded sandstone clasts (<20 mm in diameter). Two large males (∼80 kg) repeatedly walked over these specimens on this sandy matrix for 10 minutes for each bone. After the trampling exercise the specimens were reviewed for the presence of linear striations.
  Blumenschine et al. [4] did not include trampled specimens in the blind tests they reported on. However, recent debates have highlighted the difficulty experienced researchers sometimes face distinguishing trampling abrasion from cut-marks. For this reason we believed this would be an important category of modifications to include in this test [58]–[60].

Thirty six specimens were selected from the previously described aquatic butchery experiments and assembled into a single test series. Modifications were not selected on the basis of specific diagnostic micro-morphological criteria but were rather based on known activities that these specimens were exposed to (e.g. trampling). Importantly, the participants in the blind test were not given the list of possible treatments that the test specimens had undergone. In particular, we did not mention the fact that none of the specimens had been exposed to carnivore ravaging. Another important feature of the specimens included in the test series was the inclusion of bones with intrinsic marks. Aquatic bone has numerous surface vagaries that can potentially be mistaken for surface modifications. This set of specimens included five bones that had no surface modifications. However, these specimens had long linear vascular grooves which are sometimes confused with cut marks. Of the thirty-one specimens that had true surface modifications, the treatments were spread evenly across cutting and scraping actions, human gnawing, percussive activities and trampling.

Nine participants took the blind test and all of these researchers – except for one - identified modifications on all thirty six bones. Participants also provided a confidence score for each specimen diagnosis which ranged between 1 (low confidence) to 5 (high confidence). One participant (with 14 years of experience studying bone surface modifications) felt more comfortable leaving out 4 specimens from the test than identifying and diagnosing these specimens with very low confidence. The participating analysts have conducted zooarchaeological research on Pleistocene archaeofaunal collections in either eastern or southern Africa. Of these nine participants, four had at some point studied the standardized collections developed by Blumenschine and Marean [4], housed at Rutgers University. Seven of the nine participants describe themselves as zooarchaeologists, whereas two participants who did not identify themselves specifically as zooarchaeologists had collectively 17 years of experience studying experimental and archaeological bone surface modifications. All participants had at least four years of experience analyzing bone surface modifications and the experience level ranged between 4 years and 37 years of experience.

The different participants used various methodologies to determine diagnostic features of the test specimens. Five participants used just a hand lens (10–20×) to make identifications and four used both a microscope (40×) with the assistance of low incidence light as well as a hand lens.

Following the protocol of previous blind tests, ‘areas of interest’ were outlined on each specimen with a permanent marker to ensure that comparisons of identifications between analysts were associated with the same surface modification. On some specimens more than one instance of the same phenomenon was highlighted. This allowed the participants to observe limited variation in the morphology of modifications associated with a specific effector on individual specimens. Participants were asked first to identify *whether* a mark existed within the designated area and then to attribute that mark to actor and effector [61].

Participants were not given a time restriction but were urged to finish the test within thirty minutes if possible. All participants finished the test within sixty minutes. The researchers who used only a hand-lens did not take substantially less time to complete the test than those who employed a combination of microscope and hand-lens identification.

### Controlling for external influences on blind test results

A number of factors were monitored as potential drivers of variability in correspondence scores and success rates among blind test participants. Examples of these factors are:

- The inclusion of trampled specimens in the blind test: Morphological variability within experimentally trampled specimens is substantial [49]. This occasionally makes trampling abrasion difficult to diagnose confidently. Importantly Blumenschine et al. [4] included only cut-marks, tooth-marks and percussion marks in their blind tests whereas our blind test also included trampling marks. Therefore it is necessary to determine if overall correspondence levels are directly affected by the inclusion of trampling marks in the test.
- Pedagogical pedigree: Blind test participants come from a range of different pedagogical backgrounds. Consequently, they may have learnt to identify and diagnose bone surface modifications in different ways, on different collections, potentially using slightly different reference criteria. Recent debates have raised the possibility that blind-test correspondence scores may be higher between blind-test participants who learned to identify modifications within the same teaching tradition [59], [60]. If blind-tests are conducted within a group who learned within a single tradition, then we would expect to see higher correspondence relative to tests where participants derive from varied pedagogical backgrounds. Before we tried to explain differing correspondence levels through differences in external factors like instrumentation assistance type and the microstructure of marks themselves, this potential influence of pedagogical pedigree was interrogated within our dataset.
  Four of our blind-test participants have similar training backgrounds. They learned to identify and diagnose bone surface modifications on the same experimental collections (hereafter ‘TT group 1’ which comprises 6 different two-way correspondence combinations). We can therefore look at two-way correspondence scores within TT group 1 compared to correspondence amongst the other 5 participants that have different and diverse training backgrounds (‘TT group 2’ which comprises 10 different two-way correspondence combinations).
- Depth of experience: Although seven out of nine blind-test participants would call themselves zooarchaeologists, participants unavoidably had differing levels of experience with both archaeological and experimental collections. This was rounded to the nearest year.
  In looking at correspondence between, and success of individual participants it is important to note that depth of experience studying bone surface modifications varied substantially amongst blind-test participants. Depth of participant experience ranged between 4 and 37 years. One possibility that needs to be considered is whether depth of experience is an impetus behind varied success levels and correspondence between participants with equivalent levels of experience.
- Structural differences between surface modifications on aquatic bone and mammalian bone. Contingently, the need for higher magnification, greater depth of field and an applicable referential framework to accurately identify and diagnose aquatic modifications.

### Shape and size comparisons between mammalian and aquatic cut-marks

**Figure 1:**
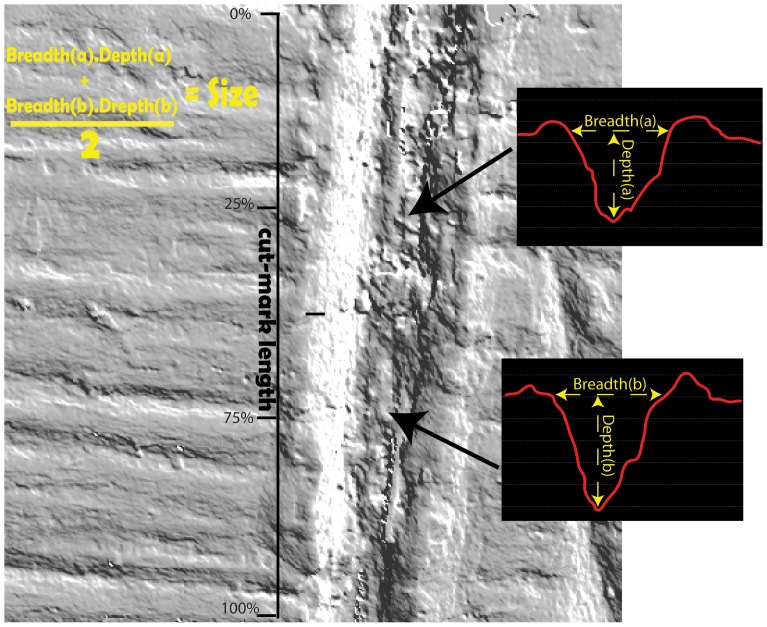
Demonstration of how mean cross-sectional size was measured on terrestrial and aquatic bone surface modifications using three-dimensional models rendered in usoft analysis software.

**Figure 2:**
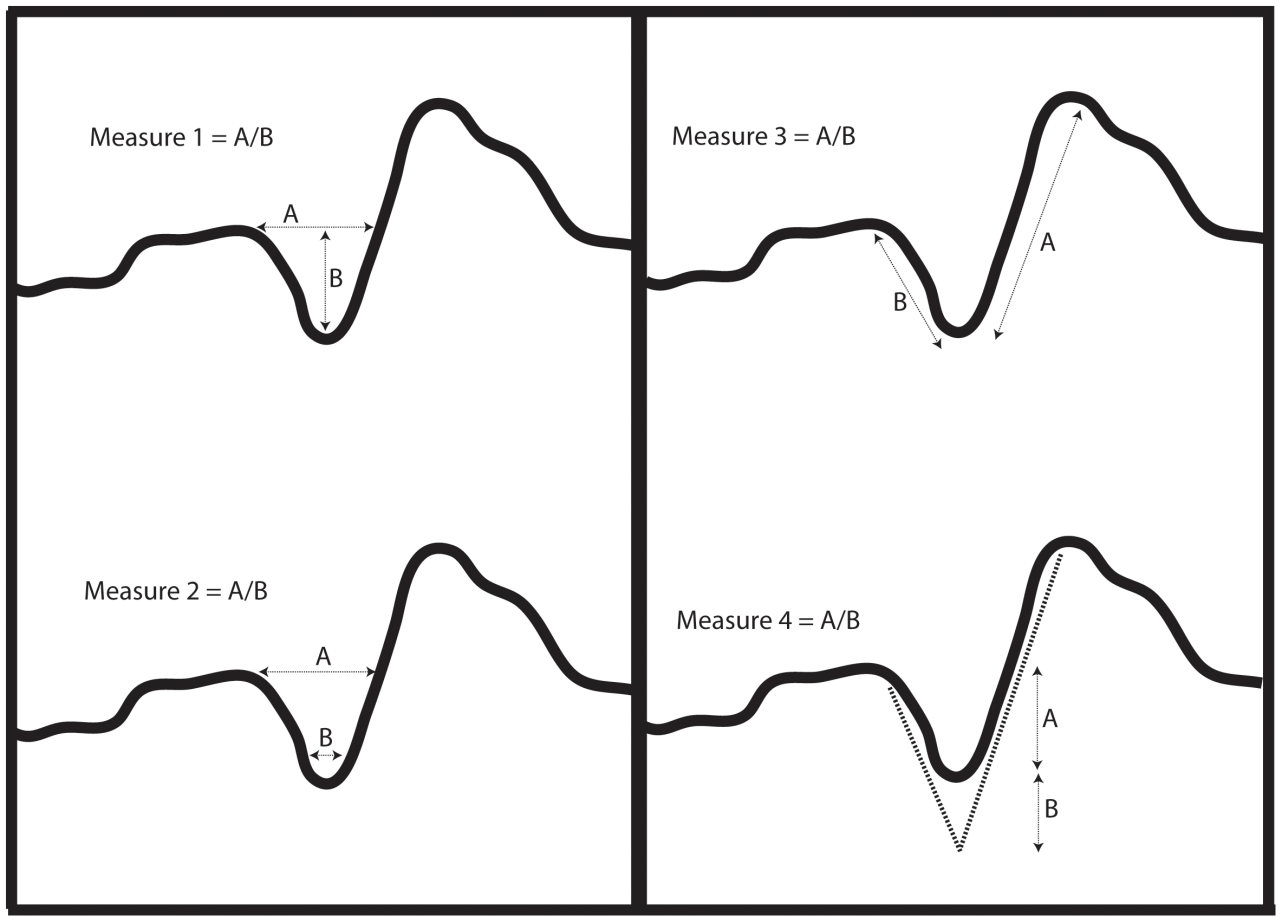
Cut-mark cross-sectional measurements 1–4 taken using three-dimensional models rendered in usoft analysis software.

**Figure 3:**
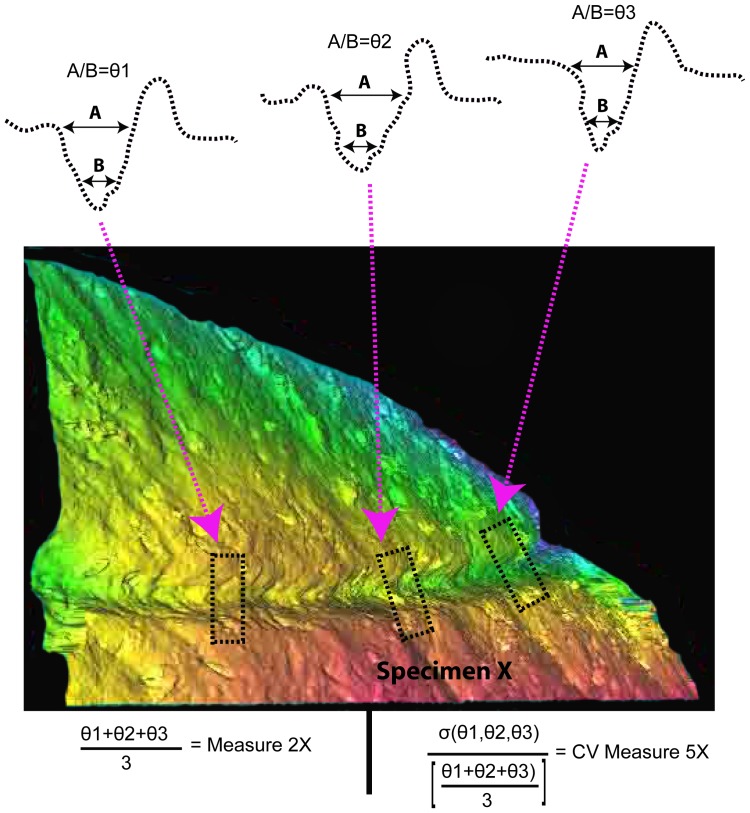
Demonstration of how measurements 5–7 were calculated.

To investigate the relative morphological differences between modifications that are produced on aquatic and mammalian bone it was necessary to take some measurements on the internal morphology of a set of measurable modifications. This was accomplished using a Nanofocus μsurf spinning disk confocal microscope which uses a high efficiency LED light source to calculate three dimensional surfaces at a resolution of 20 nm (X, Y) and has a vertical resolution (Z) of 3.1 nm. This microscope was used to generate three-dimensional models of all intrinsic and extrinsic surface features used in the blind test. We also produced three dimensional models of a large number of other modifications on both mammalian and aquatic bone not included in the blind test assemblage for measurement purposes. μsoft Analysis software was used to generate three cross-sectional profiles for each of these modifications (Figures 1,2 and 3).

We used the description of the defining features of surface modifications described by Blumenschine and colleagues [4] to guide our analysis of surface modifications. These authors specify that tooth marks have a high breadth to depth ratio and U-shaped cross-sections. In comparison cut-marks have low breadth to depth ratios for individual striae with deep V-shaped cross-sections. To focus our analysis of mark shape on these diagnostic criteria we took a series of cross-sectional ratio measurements that we believed had the potential to isolate aspects of size and shape variability associated with bone surface modification cross sections.

Ratio measurements are considered here to be the best 2D descriptors of cut-mark geometry. Absolute values as measures of cut mark shape – width, length, depth – were not selected as variables in these cross-sectional shape analyses. The use of ratio measurements factors out aspects of prehistoric butchery that are not possible to reconstruct or predict in archaeological assemblages (such as the amount of pressure that now extinct hominins would exert on tool edges and the degree of carcass desiccation prior to butchery events). The measurements were taken at 3 evenly spaced intervals on each mark (measures 2–4), apart from cross-sectional breadth and depth (measure 1) which was measured in two locations. These measures include:

- Measures 1a and 1b: Cut-mark surface breadth and overall depth. The depth measurement is taken perpendicular to the breadth measurement and was calculated from the unmodified bone surface to the lowest point on the modification. The ratio measurements used were calculated as (a) breadth divided by depth and (b) breadth multiplied by depth. Breadth multiplied by depth is used here as a proxy for overall cut-mark size (Figure 2). When used in multivariate analyses Measure 1a was normalized by the geometric mean calculated from the size measures of breadth, depth and the two shoulder heights of an individual mark (described in Measure 3).
- Measure 2: A cut-mark breadth measurement was taken at 25% of the overall cut-mark depth (measured from the base of the cut-mark upwards). The ratio measurement was calculated as cut-mark surface width (breadth) divided by the breadth at 25% of the cut-mark depth (Figure 2).
- Measure 3: Due to (1) the uneven nature of most bone surfaces and (2) shoulder effects associated with some modifications, one wall of a mark is invariably higher than the other [70]. This is so if one uses the surrounding unmodified bone surface as a plane, to orient the mark prior to measurement. Measure 3 is calculated as the height of the higher wall divided by the height of the lower wall (Figure 2).
- Measure 4: If one looks at a cut-mark in cross-section, due to their curvature the two walls rarely ever meet at a discrete point at the base of the mark itself. Through the use of image analysis tools on cut-mark cross-sections the orientation of the two walls can be projected downwards to meet at a hypothetical point below the base of the cut-mark. It is important to note here that this measure is an approximation and that its *accuracy* is influenced by the straightness of the walls of the mark being projected. If the walls are not straight, it makes their projection more difficult and consequently less accurate (Figure 2).

We predicted that bones with substantially different surface structures may influence how a tool edge behaves during the process of the actual cut-mark incision. Consequently we used three measures that we believe might reflect a portion of this variability in the context of measures 2–4 above (Figure 3).

e. Measures 5–7: these measures were constructed by calculating the standard deviation for each of the measures 2–4 for the three segments on an individual mark, and then dividing the standard deviation by the mean value for each of these measures.

Our analysis aimed at investigating (1) the mean cross-sectional size of aquatic modifications generated in our study, and (2) whether characteristics of modifications made on aquatic animals were similar in cross-sectional ratio measurements of shape to those made on large mammals. This allowed us to investigate whether the differences in modifications made on mammalian and aquatic bone surfaces were the result of differences in kind or degree or both.

### Vickers hardness tests

The differences between modifications produced on mammalian and aquatic bone may be related to variation in the structural properties of different types of bone. It is well known that mammalian bone and the bone of fish and reptiles have markedly different structures [62]. These structures appear to be related to different mechanical properties across taxonomic boundaries [63]. The exact differences in mechanical strength are dependent upon the scale of analysis. Bones that tend to be strong at the level of a whole bone may not be similarly resistant to strain and stress at the level of mineralized collagen fibrils [64]. Thus if structural properties underlie differences in bone modification frequencies it will be necessary to test aspects of the mechanical properties of bone at levels similar to the size and shape of bone surface modifications (usually <5 mm).

We chose to test the hardness of the surfaces of a variety of different types of bones using a micro-indentation technique known as Vickers hardness. This method of gauging the mechanical properties of bone employs a standardized indenter which is pressed into a flat surface of bone under a predetermined load for a specific period of time. All of our specimens were indented with a 10 kg load for 30 seconds. This process creates a depression on the surface of the bone which can be measured. The measure is directly related to the properties of the bone associated with plastic deformation of bone microstructure. As this test is related to small-scale plastic deformation we feel it is closely related to the properties that govern the reaction of a bone to tool edges. This measure of bone hardness has been related to other mechanical properties of bone such as Young's modulus [65] as well as fracture toughness [66].

There is a growing literature on the effect of various treatment of bones and how this affects their structural properties (including micro-hardness) [67]. The various treatments that affect the structural properties of bone include the nature and direction of stress and strain as well as various chemical influences on bone structure. A full review of the different impacts of various treatments is provided in [68]. We used 100 pieces of prepared mammalian and aquatic bone to compare levels of bone micro-hardness. Mammalian bones were distinguished between trabecular and cortical bone to compare variance in these two major types of bone. Individual specimens were measured multiple times because of the known intra-specimen variation in bone micro-hardness of aquatic animals [69]. Average values were calculated for specimens with highly variant signatures.

To prevent specimens from shifting during the testing phase, one surface of the bone was sheared off using a circular band saw and was ground flat with an angle grinder. The diagonals of the impression were measured to determine the Vickers hardness score. These impressions were very difficult to measure precisely from reflected light microscope images at 60× magnification. However, the use of a spinning disk laser light confocal microscope (see below for full details) allowed for very high resolution three dimensional images of the specimens. These three dimensional reconstructions were used to determine the lengths of the diagonals on the indentation. Values for Vickers hardness were calculated using the formula H_v_ = F*(1/D^2^); where F is the load applied to the specimen; D is the mean value of the diagonals of the indentation and H_v_ is the Vickers hardness value. Specimens where the full indentation was not visible were removed from the analysis. The mammalian assemblage included 13 specimens with cancellous bone. Another 39 specimens were analyzed on the cortical bone surfaces of mammalian long bones. All bones were collected from skeletons of medium to large sized bovids. The aquatic assemblage included 45 specimens that were cranial and post-cranial elements of catfish. A further 10 specimens derived from skeletons of aquatic reptiles. Unfortunately, all of the latter specimens were juvenile reptiles due to the difficulty in locating carcasses of adult reptiles. All skeletal materials had recently been macerated and therefore represented bone hardness values on “dry” bone which has been shown to have lower values than “wet” bone [63]. However, our measurements are comparisons between mammalian and aquatic bone surfaces, thus similar treatments of the two groups should provide useful comparisons. Previous assessments indicate that differences in degrees of bone moisture effects the absolute structural properties of bone but relative differences between different types of bone remains the same [63].

The relevance of using experimental deformation that occurs on dry bones as a proxy indicator for the ability of wet bone surfaces to react to incision by stone tool edges requires some inferential linkages. There is substantial literature on the capacity for experimental lab studies on dried bone to elucidate patterns in the mechanical properties of fresh bone (e.g. [63], [67]. In our experiments we measured micro-hardness values on dry bone surfaces for *all* experimental specimens. Although there are differences between the hardness values of wet and dry bone [63], the *relative* differences between different types of bone surfaces *remain constant*. Considering we used dry bone specimens for *all* the samples from which we generated micro-hardness values, we believe that our data on Vickers hardness is relevant to discussions of how different bone surfaces respond to incision by stone tool edges.

Recent tests have shown that Vickers hardness values can be affected by the orientation of the hardness indenter relative to the internal structure of the bone. As a result, all specimens were analyzed with similar orientations to the long axis of the bone. Comparisons between mammalian and aquatic bone will use non-parametric calculations of statistical significance as this variable is distributed in a manner that is significantly different from a normal distribution (Shapiro Wilk's W = .8828; p<.001).

## Results

### Blind tests

#### Identifying mark presence or absence within the designated bone surface zone

Two-way correspondence scores of hand lens assisted identifications of mark presence or absence ranged between 56% and 69% with a two way correspondence mean score between participants of 62%. However, the microscope-assisted correspondence ranged between 58% and 92%, an average of 73% (Table 3). The difference in percent correspondence between analysts in the microscope group is significantly greater than that produced by the hand lens analysts (Mann-Whitney U = 12; z = −1.921; p = .0548). If trampled specimens are removed from the blind-test (i.e. so the test includes just cut-marked, percussion marked and tooth marked specimens) the effect of instrumentation assistance type on correspondence in identifying bone surface modifications is still highly significant (Mann-Whitney U = 4.5, z = −2.74, p = 0.006). We infer that in this test of agreement on whether analysts recognized the presence of a modification, the type of instrumentation assistance used has a significant effect on the analytical outcome.

**Table 3:** Inter-analyst correspondence or agreement (number and proportion of same correct response) in (a) identifying the presence or absence of marks (locating), and (b) diagnosing the agent of modification for 36 specimens.

	Locating modifications			Diagnosing modifications	
Two way correspondence					
*Hand lens assisted*	*Abrasion included*	*Abrasion excluded*	*Hand lens assisted*	*Abrasion included*	*Abrasion excluded*
**2 and 1**	64%	63%	**2 and 1**	30%	41%
**2 and 5**	61%	59%	**2 and 5**	30%	41%
**2 and 3**	61%	60%	**2 and 3**	28%	37%
**2 and 7**	56%	56%	**2 and 7**	25%	33%
**1 and 5**	67%	70%	**1 and 5**	33%	44%
**1 and 3**	69%	71%	**1 and 3**	25%	33%
**1 and 7**	67%	71%	**1 and 7**	31%	41%
**3 and 7**	58%	60%	**3 and 7**	25%	33%
**3 and 5**	64%	63%	**3 and 5**	25%	33%
**7 and 5**	58%	63%	**7 and 5**	25%	33%
*Microscope assisted*					
**4 and 6**	58%	63%	**4 and 6**	25%	33%
**6 and 9**	78%	78%	**6 and 9**	42%	48%
**6 and 8**	75%	78%	**6 and 8**	28%	37%
**4 and 9**	67%	74%	**4 and 9**	44%	48%
**4 and 8**	67%	74%	**4 and 8**	28%	37%
**9 and 8**	92%	99%	**9 and 8**	56%	67%

Table shows results for both the inclusion and the exclusion abrasion marks associated with trampling.

Success rates for locating surface modifications were generally high. Analysts who used a hand-lens had scores ranging between 67% and 80%, with a mean score of 74%. Participants using a microscope in addition did substantially better. Their scores range between 72% and 97%, with a mean score of 88% correct. Although microscope using participants did better in locating extrinsic surface features on average, their increase in success rates relative to hand lens using participants was not significantly higher (Mann-Whitney U = 2.5, z = −1.736, p = 0.082)

#### Identifying the agent of mark production

In this second review of the blind test we investigate the frequency with which two analysts correctly diagnose the same modification. Our data reflects the correspondence between specific sets of criteria by which analysts differentiate agents of mark production. Both the *suitability* and the *replicability* of the criteria that participants used to identify modifications on aquatic specimens will also affect the degree of correspondence or commonality in the scores. For example, two analysts may have high levels of correspondence on all specimens, but if their agreement is often on specimens that are misdiagnosed, they will ultimately have low two-way correspondence scores. Thus the correspondence data refers to the proportions of only the same correct responses.

Compared to identification of the presence of marks, the correspondence of correct identification is substantially lower (Table 3). Analysts often rely on contextual criteria for diagnosing marks, particularly when micro-morphological indicators are less evident e.g. [72], [73]. All participants stated that the majority of their experience with bone surface modifications was with only terrestrial mammalian bone surfaces prior to taking this test.

The lack of familiarity of participants with aquatic butchery contexts – where and how marks are likely to occur on fish and aquatic reptiles - suggests their contextual referential frameworks had limited utility in this scenario. The trampling marks in the blind test also exhibited substantial morphological variability which may have made diagnosing agencies on certain specimens difficult.

The median value for two-way correspondence amongst analysts who used only a hand-lens was 26%, whereas the median value for correspondence between microscope-using participants was 35%. Analysts using a microscope usually had an almost 10% increase in correspondence. However, these differences are not significant (Mann-Whitney U = 16.5; z = −1.453; p = 0.146).

Success rates in identifying actors responsible for surface modifications were generally low (Table 4). The group that used only a hand-lens had accuracy rates that ranged between 36% and 47% with a median of 42%. The group that used a microscope also had relatively low accuracy scores. These ranged between 39% and 69% with a median score of 53%. This is an 11% increase in the accuracy of mark identification while using a microscope. However increase in the accuracy of mark identification associated with microscope use is not significant (Mann-Whitney U = 5; z = −1.112; p = 0.266).

**Table 4:** Individual participant success scores diagnosing modifications.

Locating modifications		Diagnosing modifications	
*Hand lens assisted*		*Hand lens assisted*	
Participant	Score		Score
1	80%		44%
2	67%		42%
3	72%		39%
5	80%		47%
7	69%		36%

*Microscope assisted*		*Microscope assisted*	
4	72%		44%
6	81%		39%
8	97%		58%
9	97%		69%

Table 3 indicates that if trampled specimens are removed from the blind-test (i.e. so the test includes only cut-marked, percussion marked and tooth marked specimens) instrumentation assistance type has an influence on the mean correspondence in diagnosing bone surface modifications, although the influence is not significant (Mann-Whitney U = 16.5; z = −1.458; p = 0.145).

Unfortunately numerous factors may influence the correspondence associated with diagnosing the agent of mark production. We investigate below (*3.4, 3.5, 3.6*) individually the influence of each of these factors already described (pedagogical pedigree; instrumentation use etc.). However there is also the possibility that there is an interaction and covariation affect between these different types of influences. To investigate this prior to individually assessing the influence of each factor we conducted a GLMM analysis using SPSS 20. In this analysis correspondence scores were identified as the dependent variable and other possible influences were incorporated as independent variables (instrumentation, pedagogical pedigree and years of experience). We found that tests of between subject effects were insignificant (years of experience f = .281; p = .600; pedagogical pedigree f = 1.475; p = .234; instrumentation f = 1.737; p = .197). Based on the results of this study we concluded that investigating the effect of each of these variables independently on correspondence scores was a more productive avenue of analysis.

### Size and shape comparisons between terrestrial and aquatic cut-marks

**Figure 4:**
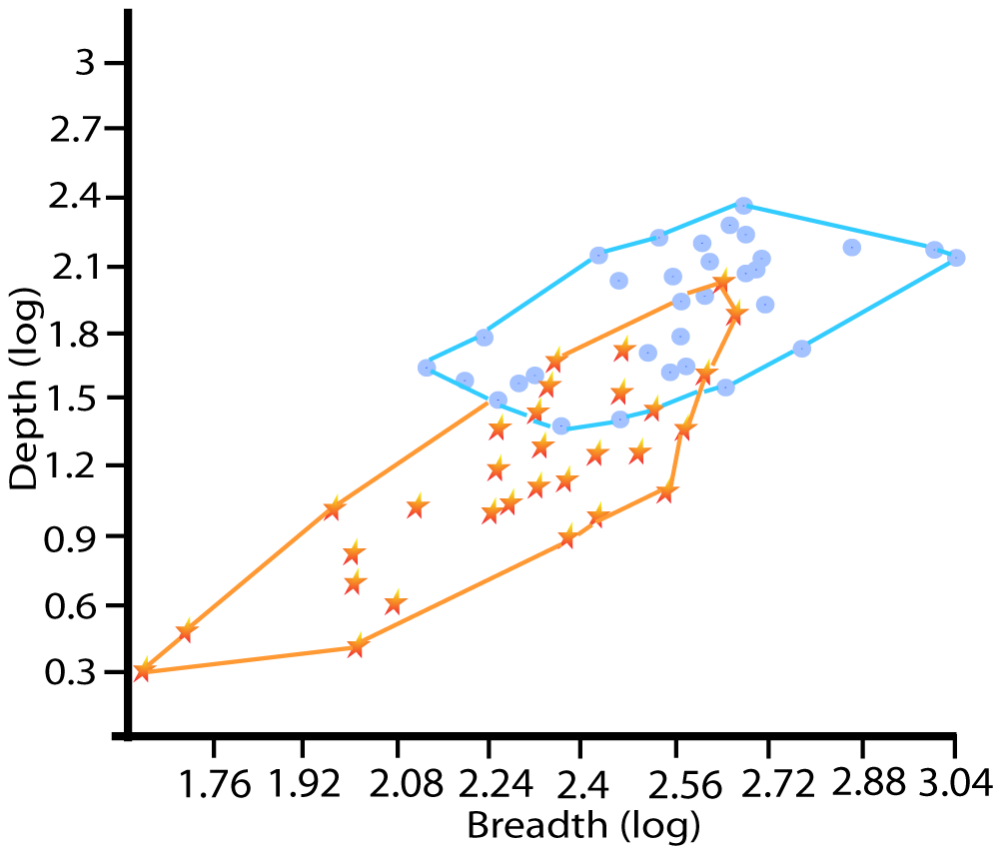
Convex hulls of cut-mark cross-sectional breadth and depth (µm) for surface modifications on aquatic (red star) and terrestrial (blue dot) bone surfaces.

**Figure 5:**
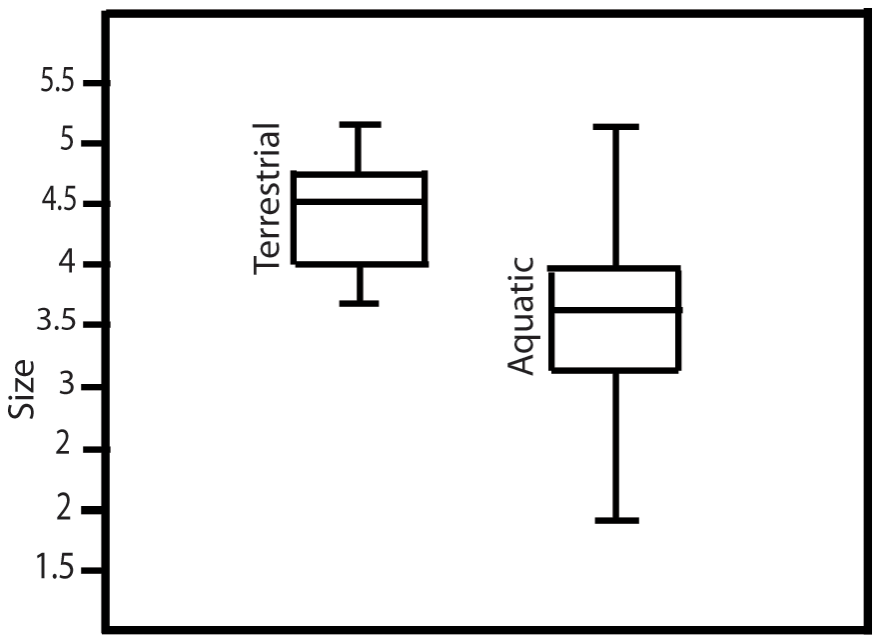
Box plot comparing cross-sectional size (log µm) for cut-marks on aquatic and cut-marks on terrestrial fauna.

Cross-sectional comparisons of aquatic cut-mark size and shape show differences between aquatic and terrestrial modifications. Cross-sectional size of modifications on the measured terrestrial specimens is significantly larger than cross-sectional size on aquatic bone surfaces (Mann Whitney U = 135; z = −4.857; p<0.001) (Figure 4 and Figure 5).

**Figure 6:**
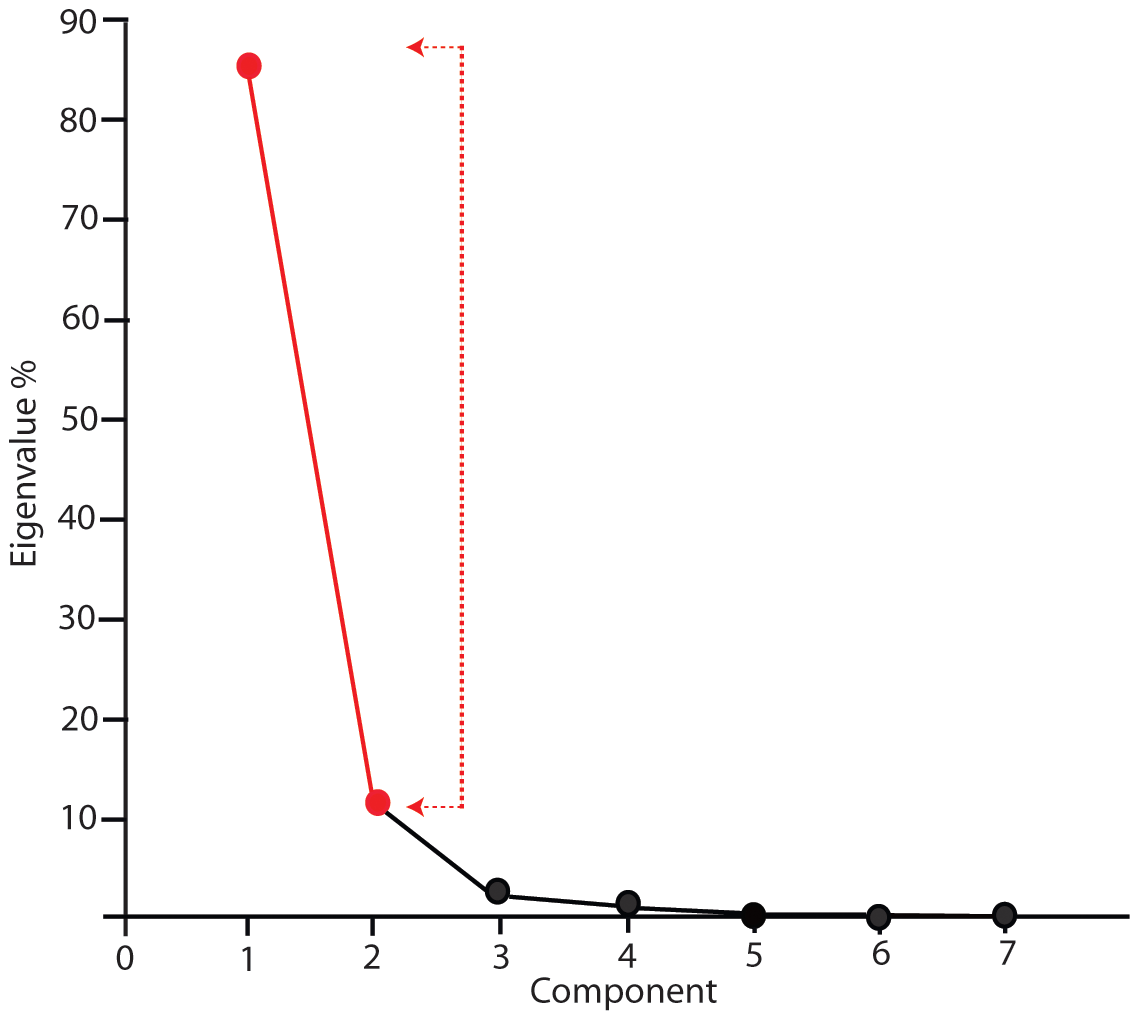
Scree plot indicating the first two plotted components in red, which account for 97.6% of the variance in the dataset.

**Figure 7:**
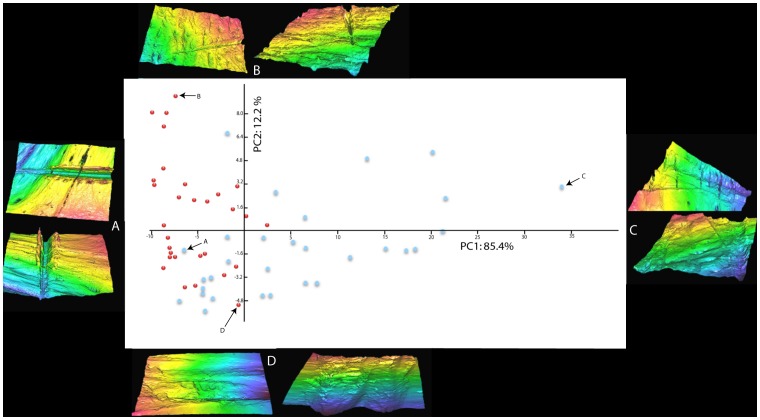
PCA using variables of measure 1a and measures 2–7. Red dots represent each terrestrial modification and blue dots represent each aquatic modification. 3D models represent within group specimens with the highest loadings on each principal component. A and C represent aquatic and D and B represent terrestrial modifications.

A principal components analysis of cross-sectional shape was conducted using the variables of measures 1a and measures 2–7. PC1 accounts for 85.4% of the variance in the dataset and PC2 accounts for 12.2% of the variance. The first two components, accounting for 97.6% of variance in the dataset were plotted (Figure 6). Figure 7 shows that the variance within cut-marks on terrestrial bone is oriented along principal component two (PC2). Measure 4 has the highest correlation with PC2 scores and is also the main driver of variability within terrestrial modifications (Table S1). However, the variance within aquatic modifications is more broadly distributed along principal component one (PC1). Measure 1a, or size adjusted breadth∶depth ratio (Blumenschine et al. 1996), has the highest correlation with PC1 scores (Table S1).

This suggests that the factors driving variance in terrestrial modification morphology (oriented along PC2) are unrelated to the factors driving variance in aquatic modification morphology along PC1. In terms of the potential influence of differences in bone surface structure between aquatic and terrestrial bone on cut-mark morphology, this is an important finding. The structural properties of aquatic bone are likely to be influencing cut-mark formation in different ways to the structural properties of terrestrial bone. Additionally, although terrestrial and aquatic modifications are not discretely clustered along PC1 and PC2, aquatic modifications are more variable and are orienting the major axis of variation in the dataset in accordance with the variance within aquatic modifications.

**Figure 8:**
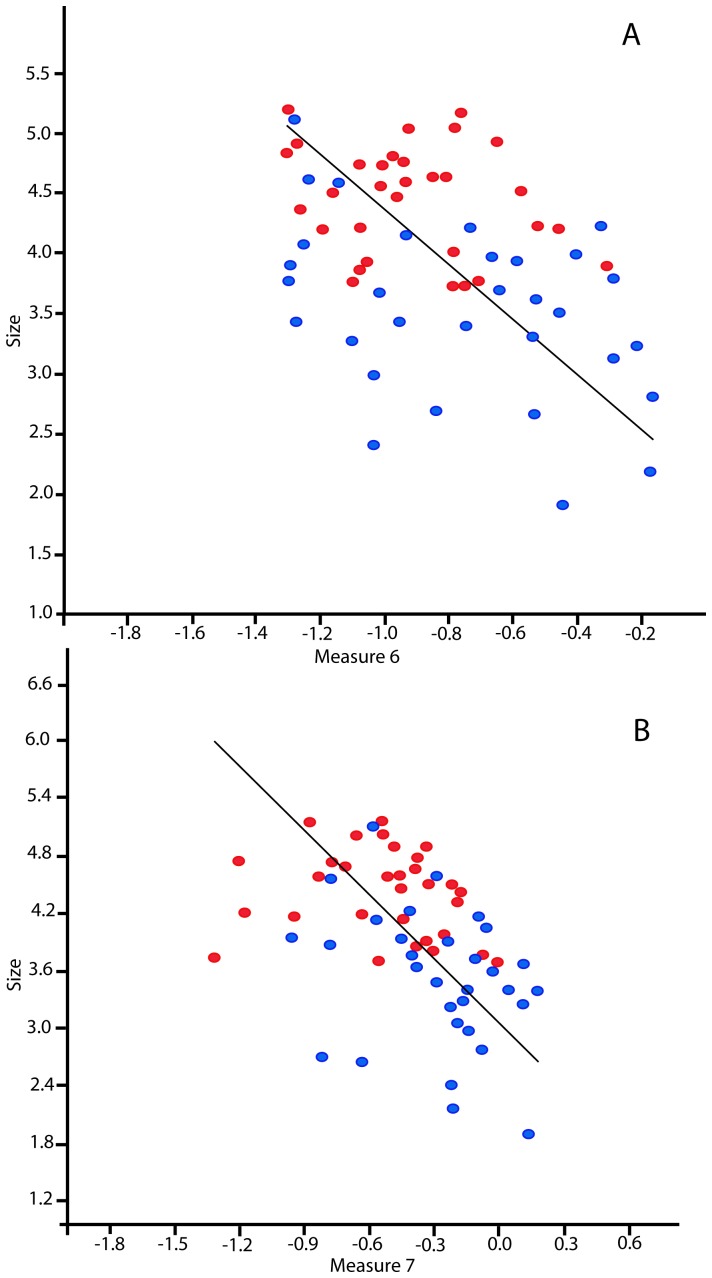
Relationships between two measures of cutmark shape and cut-mark size. (A) Regression of logged measure 6 against log size for all the cut-marks measured. Blue dots refer to aquatic modifications. Red dots refer to terrestrial modifications. (B) Regression of logged measure 7 against log size for all the cut-marks measured. Blue dots refer to aquatic modifications. Red dots represent terrestrial modifications.

Measure 6 refers to the variability in measure 3 along individual cut-marks. Interestingly size in the whole dataset is negatively correlated with measure 6. As cut-marks get smaller their morphology becomes more variable in terms of measure 6 (Kendall's Tau = −0.262, p = 0.002). Figure 8 is a regression that shows measure 6 has a negative correlation with cut-mark size (r = −0.429, p = 0.001).

Measure 7 refers to the variability in measure 3 along individual cut-marks. Size is also negatively correlated with this measure (Kendall's Tau = −0.369, p<0.001). Figure 8 is a regression that also shows measure 7 is negatively correlated with cut-mark size (r = −0.420, p<0.001).

### Success levels and surface modification size

**Figure 9:**
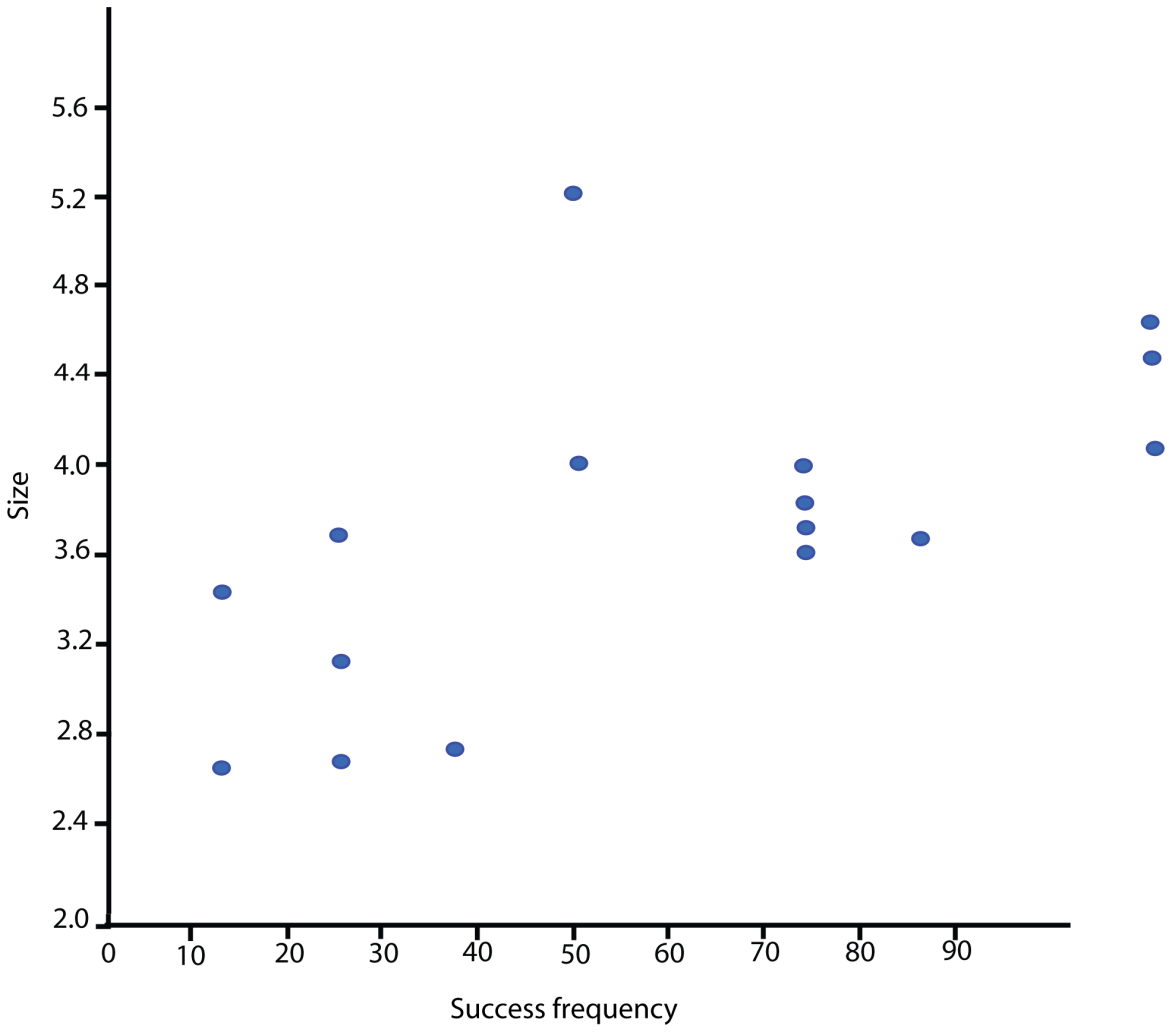
Bivariate plot showing the relationship between overall cut-mark size and the success rates of participants diagnosing modifications correctly.

In a test for correlation between blind test participant success and blind test specimen modification size (percussion marks excluded), overall participant success in *diagnosing* modifications is correlated with modification size (Kendall's Tau = 0.471, p = 0.006) (Figure 9). Hand-lens using participant success in *diagnosing* modifications is strongly correlated with modification size (Kendall's Tau = 0.538, p = 0.001). Interestingly, the success rate of participants who used a microscope to diagnose modifications is also correlated with modification size, but the relationship is not as strong as that seen in hand-lens using participants (Kendall's Tau = 0.382, p = 0.026).

In other words, as blind test modifications get larger, they get easier for participants to locate and diagnose correctly. The issue here is the initial visibility of diagnostic features rather than overall modification size. Thus, maximum dimension rather than mean cross-sectional size was chosen for comparison with participant success rates. This finding confirms the fact that modification size had an influence on the ease with which aquatic specimens were diagnosed by participants.

Mean participant confidence scores are correlated with participant success rates across the five mark types included in the test: trampling marks, cut-marks, percussion marks, intrinsic features and tooth marks (Kendall's Tau = 0.800, p = 0.050). Indeed many participants verbally identified particular specimens they struggled with and described the exact reasons why they struggled with these specimens. This suggests that participants were aware of the features that made certain specimens difficult to diagnose and this is reflected in the low confidence scores for these specimens. This finding suggests that participants' opinions on the *characteristics* of modifications on aquatic fauna that made them difficult to identify are reliable in that these difficulties are reflected in their success levels. We therefore provide a table of the general features of test specimens that participants suggested made accurate diagnosis difficult (Table 5).

**Table 5:** The characteristics of test aquatic modifications that participants suggested made them difficult to identify.

Feature	N participants who had difficulty
Overall size of the modifications	4
U shaped cross section of certain cut-marks	2
Orientation of cut-marks on cleithrum	2
Frequency of modifications on cleithrum	2
Proximity of marks on cleithrum to one another	2
Irregularity of cut-marks on clarius cranium	1
Unfamiliarity with aquatic bone surface morphology	8
Unfamiliarity with the activities associated with marks in different anatomical locations on aquatic animals	7
Unfamiliarity with aquatic modifications	8

### Influence of trampling marks on blind-test results

**Figure 10:**
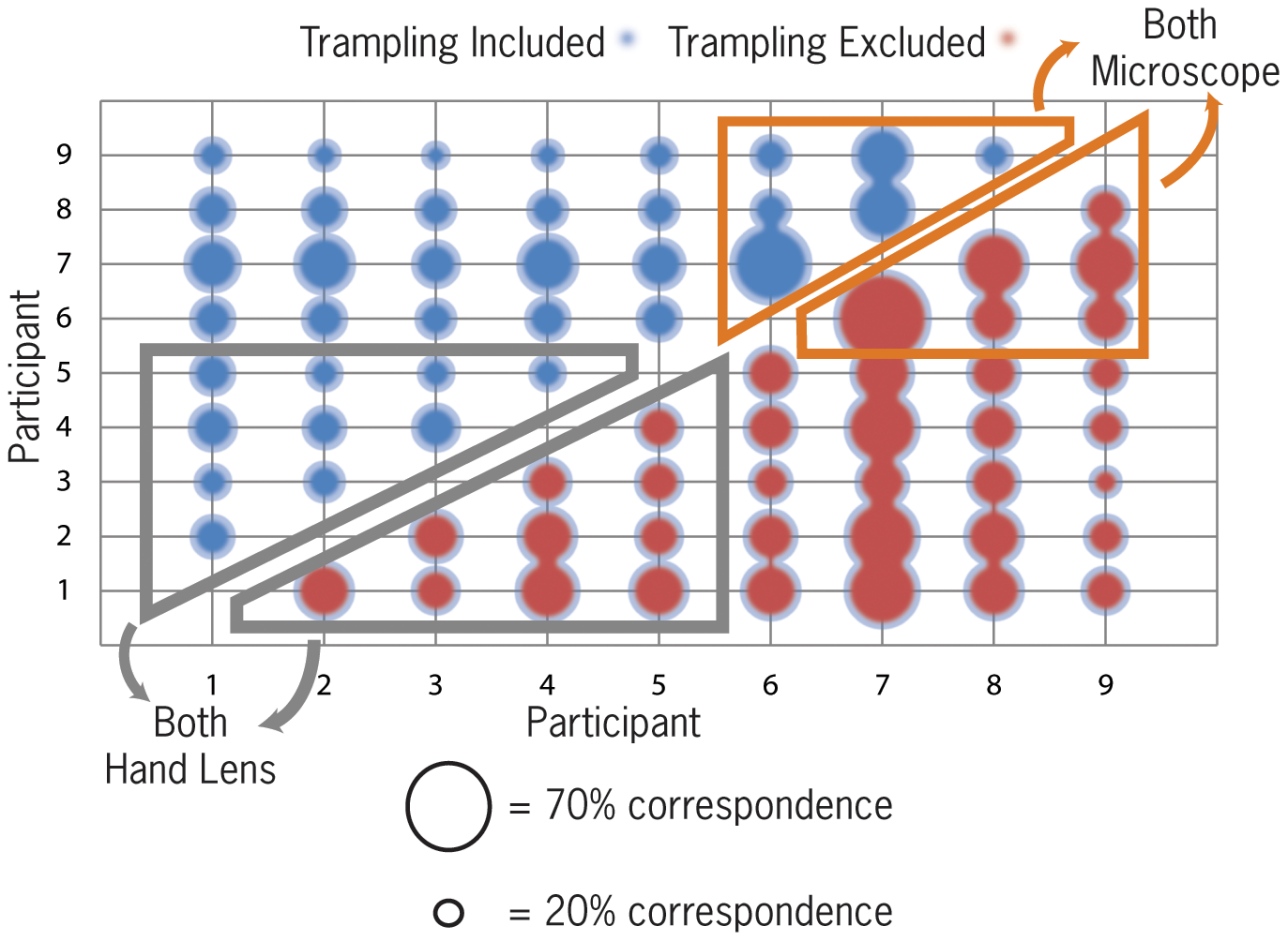
Two-way correspondence scores for participants within each instrumentation group diagnosing modifications. Correspondence scores that include trampling marks are in red and correspondence scores that exclude trampling marks are in blue.

Two-way correspondence between hand-lens using participants in *locating* modifications is not significantly increased by the exclusion of trampling marks from the test (Wilcoxon W = 34, z = 1.372, p = 0.197). However, two-way correspondence between hand-lens using participants in *diagnosing* modifications is significantly increased by the exclusion of trampling marks from the test (Wilcoxon W = 55, z = 2.848, p = 0.001) (Figure 10). Two-way correspondence between microscope using participants in *locating* modifications is not significantly increased by the exclusion of trampling marks from the test (Wilcoxon W = 15, z = 2.06, p = 0.063). However, two-way correspondence between microscope using participants in *diagnosing* modifications is significantly increased by the exclusion of trampling marks from the test (Wilcoxon W = 21, z = 2.207, p = 0.030) (Figure 10).

### Pedagogical pedigree and correspondence results

By looking at correspondence within each pedagogical group (TT groups 1 and 2 mentioned above) we can make an assessment of whether tradition and training have a significant impact on our specific blind-test results (Table 6). A Mann-Whitney test suggests that there is no significant effect of pedagogical pedigree on correspondence levels within teaching traditions in the diagnoses of modifications (Mann Whitney U = 17, z = −1.367, p = 0.171).

**Table 6:** Two way correspondence scores for individuals within the two different pedagogical groups discussed.

2-way combination	TT group 1	2-way combination	TT group 2
1 and 5	33%	9 and 8	50%
2 and 5	30%	4 and 9	39%
2 and 1	30%	7 and 9	36%
6 and 1	25%	3 and 9	36%
6 and 5	22%	7 and 8	31%
6 and 2	22%	3 and 8	28%
		7 and 4	28%
		4 and 8	28%
		3 and 7	25%
		3 and 4	25%

### Effect of experience depth on test success rates

To determine the effect of participant experience we investigated the correlation of success levels with experience depth (measured in years), for both locating and diagnosing bone surface modifications. It should be noted that the blind-test participant with 37 years of experience did do substantially better than other participants both in locating and in diagnosing test specimens. However, *overall* there was no correlation between depth of experience and success in *locating* modifications (Kendall's Tau = 0.15633, p = 0.550). Overall success in *diagnosing* modifications was also not correlated with depth of experience in our dataset (Kendall's Tau = −0.35309, p = 0.180).

The success levels of the blind test participants were fairly low relative to reported blind test results elsewhere within which only modified terrestrial specimens were used [4]. These relatively low scores in our tests can be partially explained by the lack of familiarity of all participants with modifications on aquatic bone.

### Vickers hardness tests

**Figure 11:**
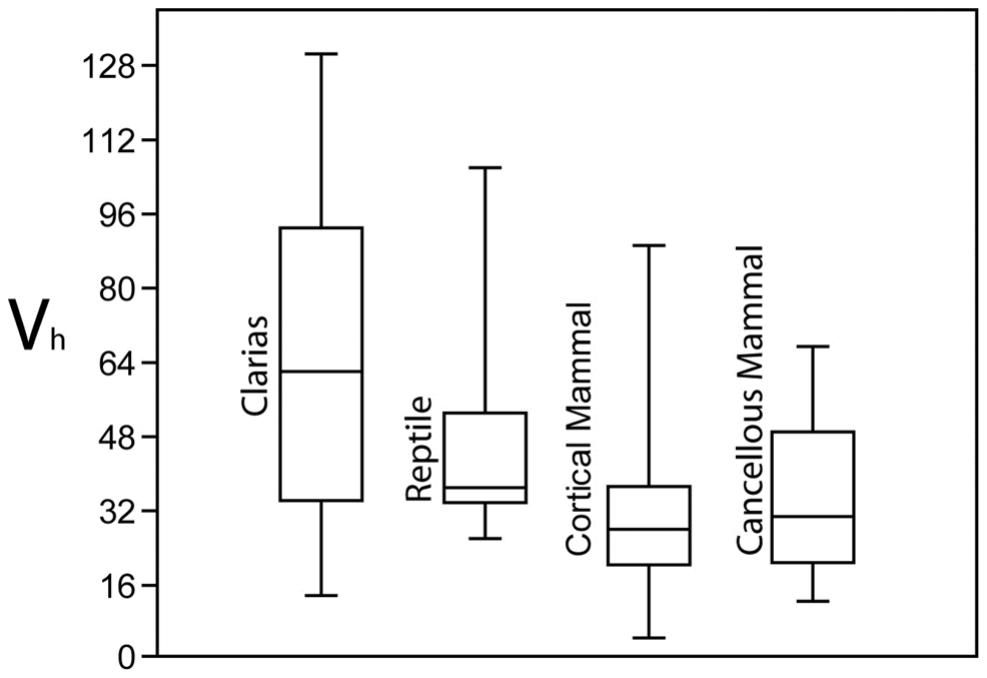
Comparison of Vickers hardness values for mammalian and non-mammalian bone. See text for tests of significance.

Vickers hardness tests documented the presence of significant differences in micro-hardness values between mammalian and aquatic modifications. Specimens were analytically separated into four groups corresponding to major bone types. These included cranial and cleithrum fragments from catfish specimens; reptilian bones; cancellous mammalian bone and cortical mammalian bone. Data indicate that these classes have significantly different Vickers hardness values (Figure 11; ANOVA; df = 3; F = 9.231; p<.001). Post-hoc pairwise comparisons of the different groups showed that the bone surfaces from the catfish were significantly harder than both cortical mammalian bone (Tukey's HSD Q = 4.409; p = .011) and cancellous mammalian bone (Tukey's HSD Q = 4.109; p = .022). Differences between reptilian bone and mammalian bone were not significant (p = .359), and differences between cancellous and cortical bone were not significantly different (p>.994). The lack of difference between reptilian and mammalian bone may be due to the fact that some of the reptilian bone tested was from younger individuals, while all other specimens were of adult individuals.

## Discussion and Conclusion

Our results show higher correspondence and accuracy levels within the sub-group of analysts that used a microscope to identify aquatic bone surface modifications. The data suggest that mark location and mark identification are both more accurate when an instrument with higher magnification and a greater depth of field is used. This indicates that degree of magnification is a relevant variable in the location and identification of aquatic bone surface modifications. This stands in contrast to evidence from mammalian bone surface modifications where inter-analyst correspondence is high when hand lenses are used [4], [74].

The fact that higher magnification and greater depth of field enables analysts to locate and identify modifications on aquatic taxa more accurately and at higher frequencies warrants some explanation. Cross-sectional size measurements of thirty randomly chosen mammalian and thirty aquatic cut-marks provide insight into why this is the case. The measurement data shows that the mean cross-sectional size of cut-marks on aquatic bone surfaces is significantly *smaller* than the mean cross-sectional size of cut-marks on mammalian bone surfaces. This smaller size of aquatic modifications indicates that diagnostic micro-morphological features characterizing these marks may also be smaller. The assistance of greater magnification makes these features more recognizable. As a result, at higher magnification test modifications were easier to recognize and diagnoses were consequently more accurate and reliable.

The ability to recognize diagnostic micro-morphological features is also particularly useful where researchers are unfamiliar with the contextual criteria associated with the production of a particular mark type. Terrestrial bone surface modification studies have extensive actualistic and experimental referential frameworks [10], [50], [71], [72], [75]–[82], by contrast aquatic referential frameworks are comparatively weak [28], [29], [14].

The analyses of cut-mark cross sectional shape identified patterns that potentially explain a portion of the blind test results. Variance in shape within the individual groups of aquatic and terrestrial bone surface modifications is oriented along different axes in the PCA. This suggests that the factors underpinning morphological variability within these two groups are unrelated. If discrete differences in bone surface microstructure between aquatic and terrestrial bone were driving cut-mark morphological variability, this is the pattern one would expect to see. This also suggests that factors underpinning the largest proportion of variability in the dataset (along PC1) are not influencing morphological variability within terrestrial modifications. This finding has direct implications for the blind-test results. It suggests that participant familiarity with the spectrum of morphological variability within terrestrial modifications (oriented on PC2 in this specific dataset) does not equip them to make accurate diagnosis on aquatic cut-marks that fall outside this spectrum.

Several of our research participants also pointed out the similarity between cut-mark clusters on the catfish cleithrum and the contextual criteria they would associate with trampling marks. In sum, the characteristics of aquatic bone surface modifications do not only represent differences of degree (e.g. smaller than marks on mammalian bone) but they are also different in the shape and context in which they are found. This makes surface modifications related to the butchery of aquatic fauna relatively difficult to identify.

The Vickers hardness tests provide an explanation for why modifications on aquatic bone surfaces are relatively small and morphologically diverse. This test was applied to a wide range of different mammalian and aquatic bone surfaces. The results indicate that the surfaces of the bones of aquatic taxa are significantly more resistant to plastic deformation than the mammalian samples we tested. Aquatic bone appears to be more resistant to deformation and it is therefore likely to react differently to the incision of a tool edge. Figures 6 and 7 indicate that shallow cut-marks generally have smaller overall dimensions. When a tool edge comes into contact with an aquatic bone surface during butchery the resultant incision is likely to be smaller – particularly shallower - than if an incision is being made with the same magnitude of applied force on a softer mammalian bone surface.

Additionally, the harder aquatic bone could have an effect on the way a tool behaves once it makes contact with the bone surface. One possibility is that higher resistance affects the ability of the tool to cut a deep straight V-shaped incision, like one would expect of a cut-mark on a terrestrial bone surface. It is possible that contact with bone could redirect the angle of applied cutting force of the tool edge when it hits the bone. This could cause the edge to “skid” and “wobble” instead of cutting into the surface with the same angle of incision as when it initially made contact with the bone surface. The effects of bone mechanical properties requires further investigation to confirm these possible relationships between bone surface properties and bone surface modification morphology.

If this was the case one could expect resultant marks to vary considerably in their cross-sectional morphology and straightness, which in consequence may make them more difficult to identify as cut-marks. The data on cut-mark cross-sectional shape can explain this to some degree. An unexpected result was that two measures we hypothesize reflect how tool edges behave during the process of cut-mark incision, are negatively correlated with cut-mark size. Indeed, as cut-marks get smaller they become more longitudinally variable in terms of cross-sectional morphology. As there are significantly more aquatic cut-marks at the smaller end of the size spectrum, these modifications are generally more longitudinally variable and consequently more difficult to identify.

The 10% discrepancy in correspondence scores in locating modifications between the group that used only a hand lens and the group that used a microscope implies an instrumentation effect on assemblage wide frequency estimates. It is difficult to compare modification frequencies between actualistic and archaeological assemblages because of the numerous biases present in archaeological assemblages. However, a discrepancy between analysts at levels of 10% would result in dramatically different interpretations of an assemblage. Archaeological assemblages that have differences in frequencies of modification upwards of 10% could be assigned to different actors of accumulation [50]. In this study, we have documented differences at similar magnitudes between analysts studying an identical assemblage of modified aquatic bones.

The additional expense and time required to analyze individual aquatic specimens under a microscope seems warranted by our results given the increased margin of identification accuracy and reliability that it affords analysts. Indeed, the acceptance of a 10× hand lens as the requirement for identification of surface modifications (as described in several studies of bone surface modification [75], [72]) may explain the infrequent documentation of aquatic surface modifications in archaeological assemblages. As a result, aquatic resources are often absent in discussions of early Pleistocene hominin dietary adaptations.

The suggestion here is not that surface modifications on aquatic faunas have been intentionally ignored by zooarchaeologists studying early Pleistocene archaeofaunas. However, the explanatory capacities of the methodologies employed have made their recognition difficult by default. Consequently surface modification studies generally rely on terrestrial records to model the subsistence behaviors of Early Pleistocene hominins. The data presented in this paper suggests that inferences of behavior based on the frequencies of surface modifications on aquatic taxa, must be approached with a different interpretive framework than that implemented for terrestrial mammals.

The remains of fish and reptiles at many early Pleistocene sites in lake shore settings are substantial. Modifications inflicted on the bone surfaces of these animals by hominins, carnivores and, inadvertently, through trampling could yield valuable additional information about the accumulation and general taphonomic history of these sites. If hominins were significant agents of modification, then identifying aquatic assemblage components can contribute substantially towards reconstructing the contexts of hominin subsistence and the specific role that aquatic resources played in fulfilling nutritional needs through time.

The paucity of actualistic referential frameworks for aquatic resource access adds ambiguity to the task of mark identification on aquatic taxa. Data on interpreting different anatomical distributions of surface modifications for fish and reptiles can, in principle, provide behavioral explanations for mark occurrence and frequency. This limitation is pertinent considering the small size of these marks often made them difficult to distinguish just on their micro-morphological characteristics alone. It has previously been documented that experience with control collections where the actor and effector are both known is a prerequisite for acquiring the ability to diagnose modifications correctly [4]. The use of similar referential frameworks may be necessary to isolate the behavioral importance of aquatic resources in early Pleistocene subsistence contexts.

Here we provide an explanation for why surface modifications on aquatic taxa may not previously have been recognized and included in reconstructions of the diets and subsistence behavior of early Pleistocene hominins. An expanded experimental framework will provide new insights into hominin dietary adaptations in ways that have not previously been explored.

## Supporting Information

Table S1 **Principal component analyses loadings of variables associated with plotted principal components 1 and 2.**
(DOCX)
