# Supplementary material for: Investigating the Signature of Aquatic Resource Use within Pleistocene Hominin Dietary Adaptations
Source: PLoS One. 2013 Aug 21;8(8):e69899. doi: 10.1371/journal.pone.0069899 (PMC3749151; doi:10.1371/journal.pone.0069899)
Supplement: Table S1 — Principal component analyses loadings of variables associated with plotted principal components 1 and 2. (DOCX) [file pone.0069899.s001.docx]

**Supporting information**

|  | PC1 | PC2 |
| --- | --- | --- |
| Measure 1a | 0.9755 | 0.2173 |
| Measure 2 | -0.03773 | 0.09104 |
| Measure 3 | 0.02551 | -0.00218 |
| Measure 4 | -0.215 | 0.9714 |
| Measure 5 | -0.00212 | 0.002876 |
| Measure 6 | 0.004959 | 0.003459 |
| Measure 7 | 0.01245 | 0.02934 |
